# Analysis of group behavior based on sharing heterogeneous roles in a triad using a coordinated drawing task

**DOI:** 10.3389/fpsyg.2022.890205

**Published:** 2022-11-15

**Authors:** Jun Ichikawa, Keisuke Fujii

**Affiliations:** ^1^Faculty of Informatics, Shizuoka University, Hamamatsu, Japan; ^2^Department of Information Systems Creation, Faculty of Engineering, Kanagawa University, Yokohama, Japan; ^3^Graduate School of Informatics, Nagoya University, Nagoya, Japan; ^4^RIKEN Center for Advanced Intelligence Project, Fukuoka, Japan; ^5^PRESTO, Japan Science and Technology Agency, Kawaguchi, Japan

**Keywords:** coordination, group behavior, social interaction, adjustment, heterogeneous role

## Abstract

Humans often share roles and aim to achieve a group goal based on sociality, which is the tendency to spontaneously involve oneself with others. Cognitive science, psychology, and neuroscience studies suggest that in such planned coordination, adjusting one’s own actions based on other roles is crucial for high task performance. However, the mechanisms of complex and dynamically planned coordination, such as non-verbal group behavior with three or more members, remain to be fully investigated. This study introduced a coordinated drawing task in a triad, quantitatively analyzed non-verbal group behavior based on sharing heterogeneous roles, and investigated an important role. Participant triads engaged in the task repeatedly by operating reels to change thread tensions and moving a pen connected to the three threads to draw an equilateral triangle. Then, the three roles (pulling, relaxing, and adjusting) had to be shared. The pulling and relaxing roles served to move the pen as if an operator pulled it closer to the hand and to support the pen’s movement, respectively. However, these roles alone could not draw a side considering the task specification. The adjusting role needed to change the tension flexibly and maintain an overall balance. In the experiment, we measured the pen positions and tensions, and established statistical models to fit the analyzed data. The results estimated that the action in the adjusting role was related to the improved performance of faster drawing on a side. This role may moderately intervene in the actions by the other roles and fine-tune without disturbing the pen’s smooth movement while avoiding great pen deviation. Our findings may suggest the crucial role as a facilitator that handles resiliently in non-verbal coordinated behavior of a triad, and contribute to our understanding of social interactions.

## Introduction

Sociality is the tendency to involve oneself with others spontaneously (De Jaegher et al., 2010; Amici and Widdig, 2019; Ichikawa et al., 2021); humans who interact based on sociality change their environments (Sebanz et al., 2006). Social interactions are observed in living organisms including non-humans and serve as a foundation for various activities. Hence, investigating the mechanisms of orderly and flexible interactions is crucial in cognitive science, which examines intelligence and behavior in society; however, these mechanisms remain to be fully understood because individuals act with many degrees of freedom and an interaction process does not uniquely converge (Kelso, 2021; Nalepka et al., 2021). In this study, we focused on non-verbal coordinated behavior in a triad and investigated a crucial role and the adjustment using an appropriate task.

Social interactions are an interdisciplinary theme studied in cognitive science, psychology, neuroscience, sports science, and biology (e.g., Tunstrøm et al., 2013; Steiner et al., 2017; Takagi et al., 2017; Hayashi, 2018; Boonman et al., 2019; van der Wel et al., 2021). In cognitive science and psychology, coordination in social interactions is classified into two categories: emergent and planned (Knoblich et al., 2011). A typical example of the former is spontaneous synchronization of body movements; individuals do not share a group goal while observing and referring to each other. Emergent coordination is linked to unintentional perceptual information processing (Schmidt et al., 1990; Richardson et al., 2007). Additionally, the dynamical systems theory is proposed; flexible group behavior emerges through adjustment by dimensional compression of the degree of freedom in each action and their mutual supplement (Riley et al., 2011; Araújo and Davids, 2016). In neuroscience, individuals’ higher motor performance is achieved when the number of others that they can refer to is greater (Takagi et al., 2019). This suggests that others’ motor information is beneficial in adjusting one’s own actions. Meanwhile, in planned coordination, which is the focus of this study, members share roles and aim to achieve an intentional group goal (Michael et al., 2020). Cognitive science studies have often observed the process of planned coordination in problem-solving and learning; smooth problem-solving without conflict and progressing positive learning are related to taking others’ different perspectives including sharing and switching roles. Such cognitive information processing is confirmed using rule discovery, mathematics, and puzzle tasks (e.g., Shirouzu et al., 2002; Battocchi et al., 2008; Hayashi and Miwa, 2009; Evans et al., 2011). These findings correspond to the distributed cognition theory (Hutchins, 1995), which explains that an overall group function works through interactions based on relationships among subsystems where each subsystem is considered a role. A sports science study similarly indicated that cooperative and defensive group behavior functioned as one system in a basketball game composed of switching and overlapping hierarchical roles, depending on the emergency level (Fujii et al., 2016). Furthermore, psychology studies explain that representing roles, monitoring, and anticipating actions of one another are needed for coordination (Sebanz et al., 2003, 2006; Sebanz and Knoblich, 2009). Monitoring may be like referring to others’ motor information as mentioned above in emergent coordination.

In such ways, adjustment based on other roles is crucial for achieving a group goal or high task performance; however, complex and dynamically planned coordination, such as non-verbal group behavior with three or more members, is not yet fully investigated. Complexity suggests that explaining and modeling the interaction is harder in a triad than in a pair (Yokoyama and Yamamoto, 2011). Relationships among members diversify and their patterns do not converge uniquely. Many cognitive science and psychology studies assume pair interactions (e.g., Shirouzu et al., 2002; Hayashi and Miwa, 2009; Böckler et al., 2012). Laboratory experiments are conducted to control environments and identify factors that influence performance and pair interaction processes. On the other hand, dynamic features are recorded as non-verbal and time-series data, such as body movements (Braun et al., 2009). For example, some studies introduce pair motor tasks and aim at expanding classic game theory proposed by discrete selection tasks. These works analyze time-series data of upper limb movements during the tasks (Braun et al., 2009; Chackochan and Sanguineti, 2019). Therefore, quantitative analysis of the group behavior with three or more members and discussion of relationships between achieving a group goal, coordinated group behavior, and members’ roles would lead to our understanding of social interactions; however, few such studies have been conducted.

This study introduced an experimental task in a triad, analyzed the non-verbal group behavior based on sharing roles, and investigated a crucial role for high task performance. To understand complex and dynamically planned coordination, it is necessary to consider an appropriate task that satisfies the following two conditions (Ichikawa and Fujii, 2020): (1) Group behavior is controlled, which indicates that a group goal is clear, and each member’s action is based on the task rules. For example, in our previous study (Ichikawa et al., 2021), which analyzed children’s group behavior during nursery activities, it was difficult to fully discuss cognitive information processing related to the feature, as children spontaneously and freely interacted with others. Additionally, it is hard to pursue the generality of these findings, as children’s data in nursery are precious and few. (2) Cognitive information processing is reflected in the recorded body movements. In this case, it suggests that a strange action is exhibited if a player does not understand others’ or one’s own roles.

**Figure 1:**
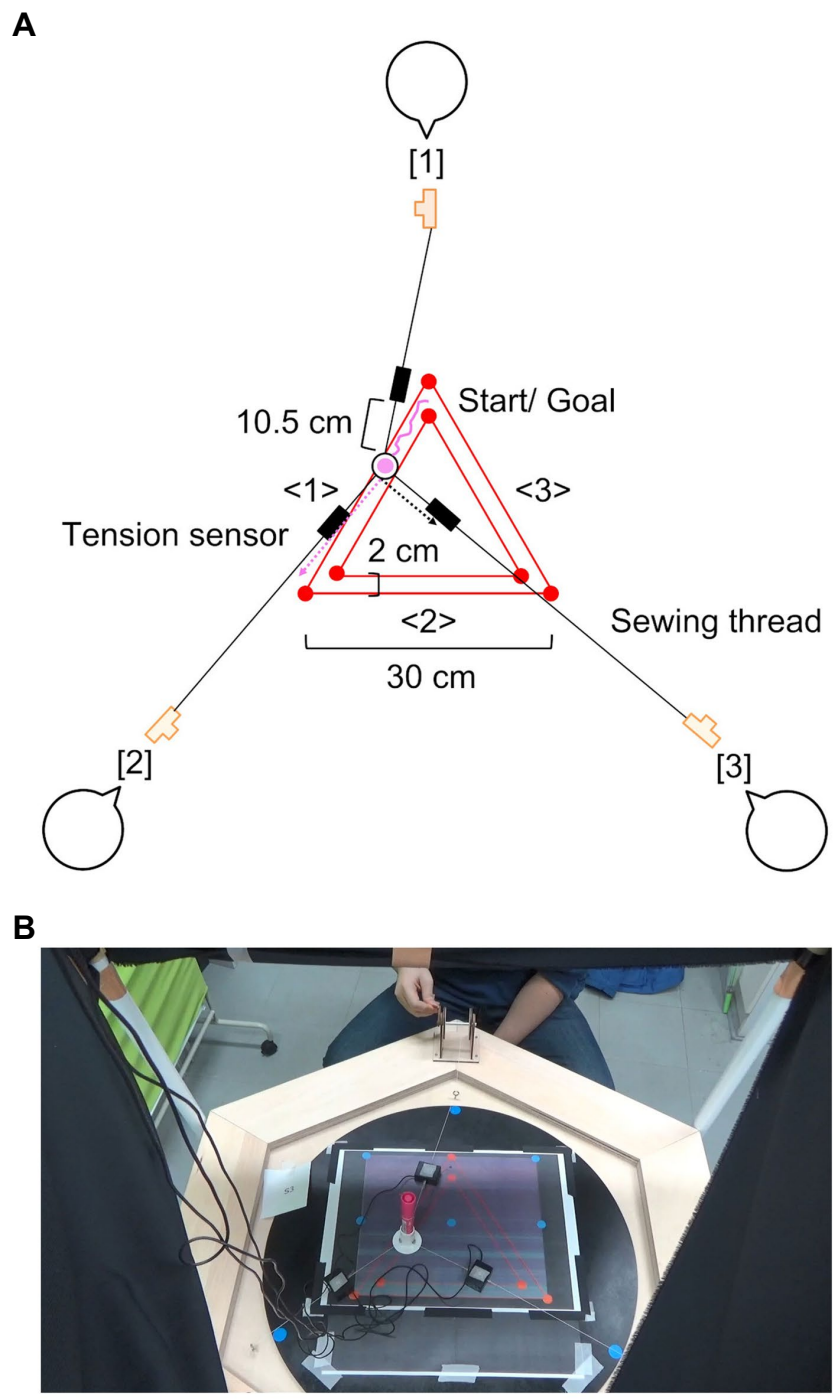
Coordinated drawing task. **(A)** Represents the pattern diagram when drawing side <1>. The length and width of each triangle’s side are 30 cm and 2 cm, respectively. Then, if operator [3] in the adjusting role does not generate any tension, the pen greatly deviates from the outside, shown by the dotted magenta arrow. Conversely, if the operator pulls the pen excessively, it greatly deviates from the inside, shown by the dotted black arrow. **(B)** Represents the experimental image. It was recorded from a bird’s-eye view using a video camera. The origin source of this figure refers to our proceedings paper (Ichikawa and Fujii, 2021).

Here, we used a coordinated drawing task in a triad where a triad operated reels to change thread tensions based on sharing the three heterogeneous roles (pulling, relaxing, and adjusting) and move a pen connected to the three threads to draw an equilateral triangle (Maruno, 1991, Figure 1, see the Coordinated Drawing Task section). The pulling and relaxing roles served to move the pen as if an operator pulled it closer to the hand and to support the pen’s movement, respectively. However, these roles alone could not draw a side, as shown by a dotted magenta arrow in Figure 1A. The adjusting role needed to change the tension flexibly and maintain overall balance to the extent that the pen did not greatly deviate from the inside, shown by the dotted black arrow in Figure 1A. This study focused on the third role because such a role is required regardless of group activity type. In learning and debate studies, a facilitator who promotes smooth and active interaction is important (e.g., Berta et al., 2015; Lessard et al., 2015). Therefore, we hypothesized that the adjusting role as a facilitator was crucial for high task performance in the coordinated drawing task. In the experiment, the pen positions and tensions were measured, and statistical models were established to fit the analyzed data. To investigate which roles were related to improved performance, we estimated the relationships between (1) performance and trial, and (2) improved performance through trials and three-role actions.

The novelty and methodological contribution of our work develops the range of discussion on planned coordination to non-verbal group behavior in a triad; it may primarily contribute to elaborating or expanding the theories applied to problem-solving and learning in cognitive science. Moreover, for effective collaborative work and the development of supporting computer systems (e.g., Zhu and Zhou, 2006; Zhu, 2021), our findings may support the need to prepare the modeling of a flexible role, which handles resiliently to help others. In the future, this study may also be adapted to predict degrees of coordination using multiagent and AI systems.

## Materials and methods^1^

### Coordinated drawing task

This study used a coordinated drawing task (Maruno, 1991), where a triad operates reels to change thread tensions and move a pen connected to the three threads to draw an equilateral triangle (length: 30 cm, width: 2 cm, Figure 1; see the Supplementary movie). The thread is pulled or relaxed when each operator turns the reel inward or outward. The triad’s goal is to draw directly without deviating from the length and width of each side. During the task, the triad should share the three heterogeneous roles (pulling, relaxing, and adjusting) and switch them counterclockwise when the drawn side changes. Table 1 represents the three roles each side in Figure 1A. We can investigate complex and dynamically planned coordination, as the group goal, rules, and roles are clear, and these features allow us to observe controlled group behavior. Additionally, we can analyze task performance and the three-role actions quantitatively by measuring the pen positions and tensions.

**Table 1:** Three heterogeneous roles required in the coordinated drawing task (Figure 1A).

Side	Operator		
	[1]	[2]	[3]
	Relax	Pull	Adjust
	Adjust	Relax	Pull
	Pull	Adjust	Relax

### Participants

Eight triads (four male and four female triads) consisting of 24 participants engaged in the coordinated drawing task. Preparing triads that were as uniform as possible was essential to investigate the effects of trial and role sharing on task performance. Hence, same-age university students participated in the experiment. Furthermore, all were right-handed, and knew and often talked with each other prior to their participation. Two triads (one male and one female) were excluded from all the analyses because they did not engage in the task according to the experimenter’s instructions. The average group age of the remaining six triads was 20.78 years (*SD* = 1.31). For reference, a pre-experiment survey was conducted to confirm the intra-individual factor that might influence their interactions and task performance. This study used a subscale of the Japanese version of the Interpersonal Reactivity Index (IRI) to measure each participant’s perspective-taking ability (Himichi et al., 2017), containing seven questions measured on a 5-point Likert scale (where 1 = Not well at all and 5 = Very well). After converting the points of two reverse items, the score range was adjusted from 0 to 28. A higher score indicated a higher perspective-taking ability. The average group score was 17.28 (*SD* = 1.67). No triad recorded an average group score outside the ±2*SD* range; this ability was regarded as being homogeneous between the triads to some extent.

We explained to the participants how we would video-record and collect data. Written informed consent from all was obtained. This study was approved by the ethics and safety committee of Kanagawa University, where the experiment was conducted and where the first author was affiliated at that time. Our work was carried out following all mandatory regulations. According to the informed consent, experimental images do not contain identifying information.

### Procedure and environment

The experimenter instructed the participants that the group goal was to move a pen by operating each reel and to draw an equilateral triangle directly without deviating from each side’s length and width. They were instructed on how to use the reels to pull or relax the threads but were not instructed on the details of the three roles. The time limit for drawing three sides was 90 s per trial. The rules of the pen’s start and goal positions and the direction to draw counterclockwise were the same across all trials. A practice trial was conducted without the time limit. After the practice, each triad repeated the task for 20 min per session. Three sessions were conducted with a 5-min break between sessions. Conversations and gestures were prohibited during each session.

**Figure 2:**
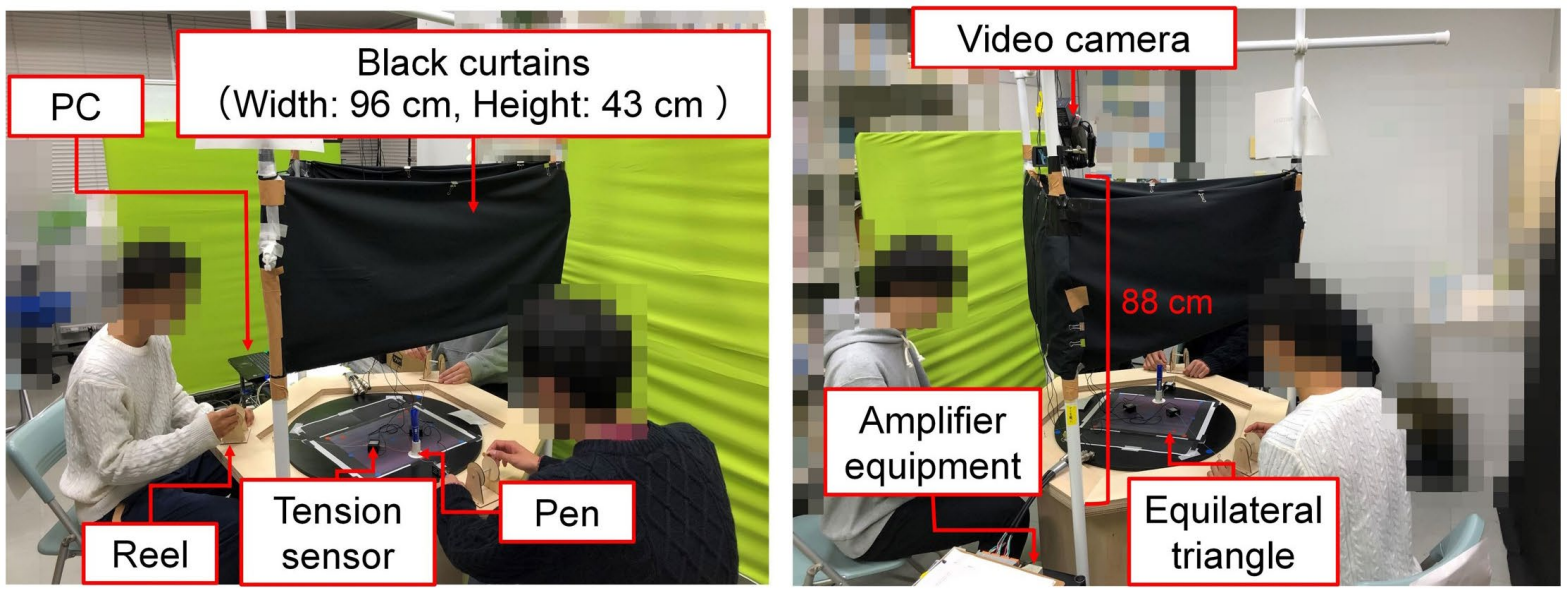
Experimental environment. The left image shows the right and left-side participants correspond to operators [1] and [3], and the right image presents the left-side participant corresponds to operator [2] in Figure 1A. According to the informed consent, all the images are shown while blurring some sections to avoid identifying individuals. The origin source of the figure refers to our proceedings paper (Ichikawa and Fujii, 2021).

Figure 2 shows the experimental environment. Each thread tension in one dimension was recorded on a personal computer (Panasonic Corp., Let us note *CF*-SX3) at 100 Hz using three sensors (Tokushukeisoku Co., Ltd., TK-440-01 in TK-A-30 N type), amplifier equipment (KYOWA Co., Ltd., PCD-300B), and dedicated software (KYOWA Co., Ltd., DCS-100A ver. 04.43). A positive value (N) was recorded in response to tension when a reel was turned inward, and the tension decreased when it was turned outward. Black curtains were placed in front of the participants so that they did not see each other’s facial expressions and make eye contact as much as possible. The task activities of each trial were recorded from a bird’s-eye view using a video camera (Sony Corp., HDR-CX680, Figure 1B). The video images (width: 1280 px, height: 720 px) were automatically digitized using motion analysis software (DITECT Co., Ltd., DIPP-Motion V/2D ver. 1.1.31) to capture the pen positions in two dimensions at 20 Hz (see the Supplementary material to refer to the measurement errors including the tensions).

## Analysis

### Task performance

We analyzed the two indices of task performance: (1) the degree of pen deviation on a side (cm) and (2) the time taken to draw a side (s). These values were calculated using the following equations:


(1) $$Dev_{i_{\left(f\right)}}=\frac{1}{\left\|P_{ver_{\left(i+1\right)}}-P_{ver_{\left(i\right)}}\right\|}|\begin{array}{c} P_{ver_{\left(i+1\right)}}-P_{ver_{\left(i\right)}}\\ P_{pen_{\left(f\right)}}-P_{ver_{\left(i\right)}} \end{array}|\text{,}$$

(2) $$Dev_{min_{\left(f\right)}}=\min\left(Dev_{i_{\left(f\right)}}\right)\text{,}$$

(3) $$\bar{Dev_{i}}=\frac{1}{F}\sum_{f=1}^{F}Dev_{min_{\left(f\right)}}\text{.}$$

where $P_{ver_{\left(i\right)}}=$($x_{ver_{\left(i\right)}}$, $y_{ver_{\left(i\right)}}$) and $P_{ver_{\left(i+1\right)}}$= ($x_{ver_{\left(i+1\right)}}$, $y_{ver_{\left(i+1\right)}}$) are the two fixed vertex positions of the triangle with a range of 1 ≤i≤3. If positive integer i=3, then $P_{ver_{\left(i+1\right)}}$ indicates the return to $P_{ver_{\left(1\right)}}$. The line connecting these points represents the median width line on side <i>. $P_{pen_{\left(f\right)}}$= ($x_{pen_{\left(f\right)}}$, $y_{pen_{\left(f\right)}}$) represents the pen position in the current time frame f. We calculated the distances between the pen position and each median line, and the minimum value $Dev_{min_{\left(f\right)}}$. The index of the time taken to draw a side represents that taken to change firstly the combination of i and i+1 when calculating $Dev_{min_{\left(f\right)}}$. The number of time frames on a side F was determined by this procedure, and the average pen deviation on a side $\bar{Dev_{i}}$ was analyzed as another index. For example, in Figure 1A, the combination to calculate  $Dev_{min_{\left(f\right)}}$changes from i=1 and i+1=2 to i=2 and i+1=3 when the drawn side switches from <1> to <2>. Smaller values of both indices indicate faster drawing without deviating from each side.

### Role actions

The degree of tension (N) was recorded by three sensors through turning reels inward and pulling the threads. We used the time-series data to analyze the three-role actions. A low-pass filter was applied to the data at 0.5 Hz to remove high-frequency noise. Additionally, a threshold value of 0.2 (N) was applied to extract at least one tension peak in each sensor. This study analyzed these tension peak data according to the following procedures.

**Figure 3:**
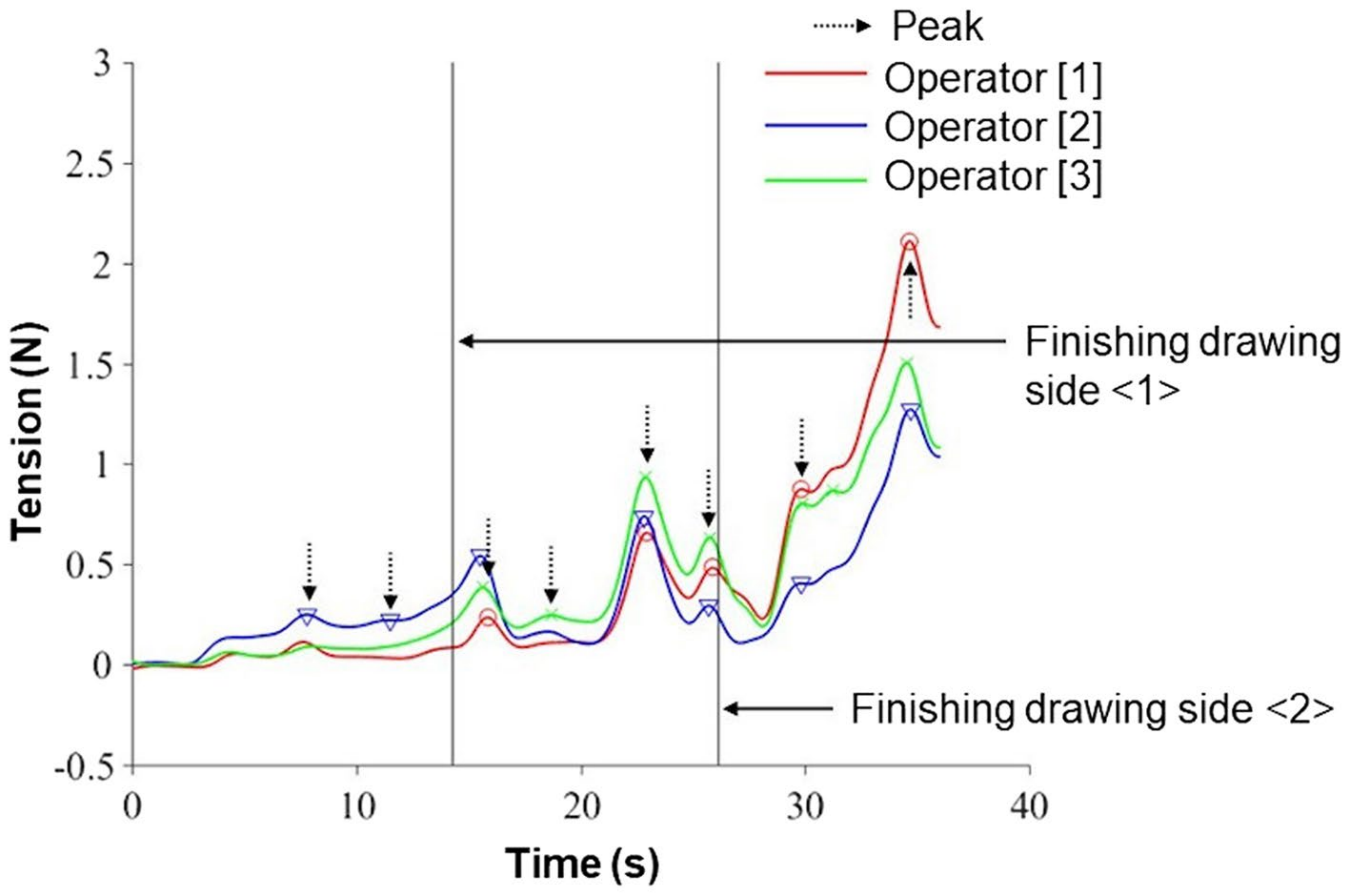
Pattern diagram of the analysis procedures for the pulling-role actions in Table 1 (see the “Role Actions” section). In this trial, the number of counts (defined as peak frequency) recorded tension peaks are 2, 4, and 2 on each side, shown by the dotted black arrows. These tensions corresponding to the vertical axis are averaged on each side, and it is regarded as the peak value. The same analysis was applied to those in the adjusting role. Meanwhile, in the relaxing role, the minimum one corresponding to the vertical axis was used on each side as the peak value considering the task specification.

After identifying a drawn side, the window length was set as the time frames taken to draw a side, and the number of tension peaks was counted and defined as peak frequency. Furthermore, we also investigated the other aspect in the three-role actions. Within the range mentioned above, the tension peak values (N) were averaged on each side in the pulling and adjusting roles. It indicated how strongly the participant pulled the pen. These peak values would provide richer information on reel operations. Meanwhile, in the relaxing role, the minimum peak value on a side was used because this role was not required to increase the tension and pull the pen considering the task specification. Figure 3 presents the pattern diagram of these analysis procedures for the pulling-role actions according to Figure 1A. The peak frequency was 2, 4, and 2 on each side, as shown by the dotted black arrows. These tensions were averaged on each side, and it was regarded as the peak value. The same analysis was applied to those in the adjusting role. In the relaxing role, the minimum tension peak was used on each side. A lower peak frequency was desirable because it would be related to the pen’s smooth movement; tension peaks might suggest characteristic reel operations. For the adjusting role, which was the focus of this study, these features might indicate explicit handling of some mistakes in operation, including role switching.

All the analyses including task performance were conducted using MATLAB R2016b. There were missing data from two trials from one triad and three trials from another due to measurement problems, such as thread breaking.

### Statistical modeling

#### Relationships between task performance and trial

This study analyzed whether task performance was improved through trials. Here, a linear mixed model was introduced to fit the analyzed data and investigate the average relationships between performance and trial considering the variabilities between triads and sides. We referred to a tutorial paper (Brown, 2021) and selected well-fitting models according to the following procedures (Supplementary code 1).

The index of the degree of pen deviation on a side (cm) or the time taken to draw a side (s) was set as the dependent variable. The trial was the fixed effect of the independent variable, and the six triads and three sides were regarded as the random effects. Then, we established two models: the intercept and full. The former included the random effects only in the intercept of a regression. It indicated that the intercept varied between the triads and sides. The latter included the random effects in both slope and intercept, which varied between the triads and sides. After estimating these regression parameters, a likelihood ratio test using the anova was conducted to compare the two models and investigate the effect of the random slope. Additionally, we calculated the Akaike Information Criterion (AIC) values. Finally, a well-fitting model was selected, satisfying the criteria that a significant difference was confirmed by the statistical test at the 5% level and the degree of freedom was larger than zero, not indicating a saturated model, and the AIC value was relatively small.

Meanwhile, before the regression analysis, the statistical tests were conducted on the time-series data of task performance through trials to investigate pseudo correlations between the dependent and independent variables (Supplementary material).

#### Relationships between improved task performance and role actions

According to the results mentioned above, the average relationships between improved task performance and role actions were estimated by a linear mixed model. We referred to the tutorial paper (Brown, 2021) and selected well-fitting models based on the following procedures (Supplementary code 2).

If the fixed-effect slope of the trial was significant at the 5% level, this performance index was regarded as the dependent variable. The three actions involved in the pulling, adjusting, and relaxing roles were used as the fixed effects of the independent variables, and the six triads and three sides were regarded as the random effects. We prepared the two indices of role actions: the number of counts recorded tension peaks (defined as peak frequency) and the peak value (N) (see the Role Actions section). The peak frequencies or values in the three heterogeneous roles were the fixed effects of the independent variables. It should be noted that all values in each independent variable were centered on the average to avoid multicollinearity as much as possible. Additionally, interactions between the independent variables were not considered, as it was difficult to interpret group behavior. We established the intercept and full models to fit the analyzed data. Before estimating these regression parameters, we calculated the Variance Inflation Factor (VIF) value of each independent variable in each model. If the VIF value was greater than the criterion 10 (Zuur et al., 2010), the independent variable would be influenced by multicollinearity. Hence, the variable was excluded, and the model was corrected. After the estimation, we calculated the AIC values, and a well-fitting model with a relatively small value was selected.

All the statistical analyses by a linear mixed model were conducted with R-3.6.1, using the lme4 1.1–26 and lmerTest 3.1–3 packages. The car 3.0–13 package was also used to analyze the tension peak values by ANCOVA to investigate the effects of the triad and side factors in each role (Supplementary code 3).

## Results

### Tension peaks in role actions

**Figure 4:**
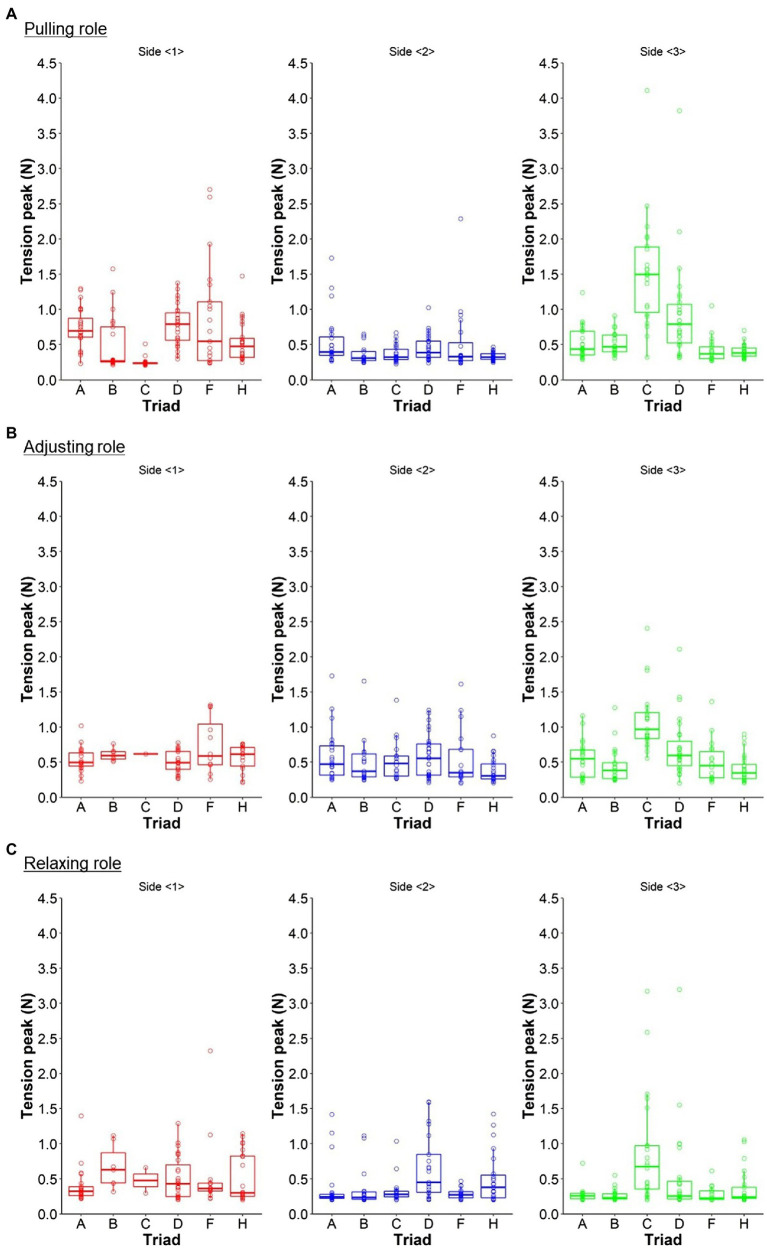
Box-and-whisker chart and plot of the tension peak value (N) of each trial on each side in each role. **(A,B)** Show the average values in the pulling and adjusting roles, respectively. **(C)** Represents the minimum one in the relaxing role. Triads E and G were excluded from all the analyses, as they did not engage in the task according to the experimenter’s instructions. For reference, these tensions vary between the triads or sides.

Six triads engaged in the task for an average of 24.5 trials (*SD* = 2.06). The tension peak value (N) of each trial on each side in each role is plotted in Figure 4; in the relaxing role, the minimum ones are used. The box-and-whisker charts are also shown.

We conducted ANCOVA to investigate the effects of the triad and side factors in each role; the tension peak value (N) in each role was regarded as the dependent variable, and the triad and side were the independent variables. ANCOVAs indicated that in all the roles, the main effect of the triad factor was significant (Pull: $F\left(5,407\right)=8.144,p<.001\text{;}$ Adjust: $F\left(5,322\right)=9.149,p<.001$; Relax: $F\left(5,335\right)=5.254,p<.001)$. In the pulling and adjusting roles, the side factor was also significant (Pull: *F*(2,407) = 20.934, *p* < .001; Adjust: *F*(2,322)= 3.679, *p* = .026; *F*(2,335)= 1.749, *p* = .176). Meanwhile, in all the roles, the interaction between the triad and side factors was significant (Pull: *F*(10,407) = 15.648, *p* < .001; Adjust: *F*(10,322)= 4.457, *p* < .001; Relax: $F\left(10,335\right)=3.639,p<0.001$).

These results might suggest the meaning of investigating the relationships between task performance and trial, and improved performance and role actions considering the variabilities between triads and sides. However, it should be noted that ANCOVA is conducted under the assumption that there are no interactions (Leppink, 2018). According to the results, we could not ignore the interaction effects; this study treated them for reference.

### Estimation of relationships between task performance and trial

**Figure 5:**
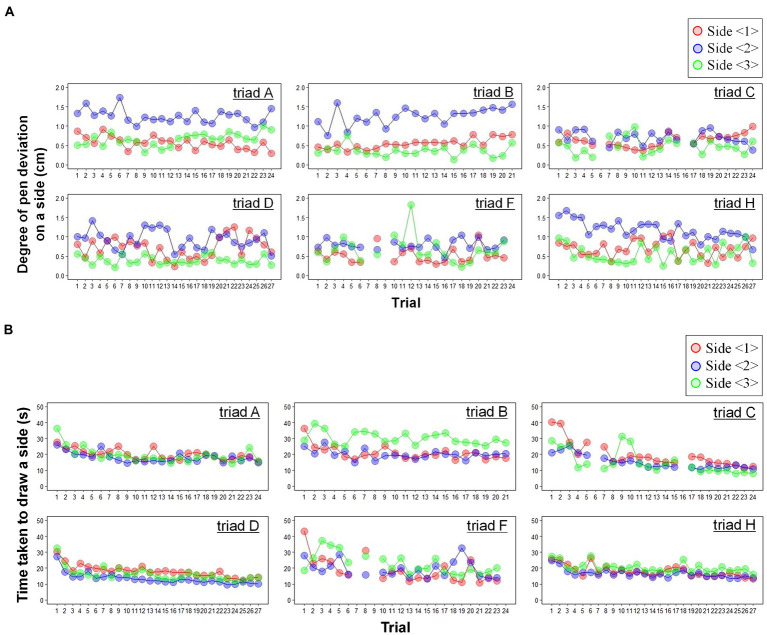
Time series of task performance. **(A,B)** Show the degree of pen deviation on a side (cm) and the time taken to draw a side (s) through trials, respectively. There are missing data due to measurement problems, such as thread breaking. The triads drew the triangle quickly through trials while maintaining a certain degree of pen deviation.

Figure 5 shows two time series of task performance: (A) the degree of pen deviation on a side (cm) and (B) the time taken to draw a side (s) through trials (see the Supplementary statistical results to confirm the results of the pseudo correlations). We analyzed the relationship between each performance and trial considering the variabilities between triads and sides by a linear mix model.

Table 2 represents the estimated relationships in the well-fitting models (Supplementary material). The full models were selected in both performance cases (*Deviation*: $\chi^{2}\left(4\right)=9.858,p=0.043,\;\mathrm{AI}C_{f}=36.453,\mathrm{AI}C_{i}=38.311\text{;}$ *Time*: $\chi^{2}\left(4\right)=22.201,p<.001,\;\mathrm{AI}C_{f}=2464.3,\mathrm{AI}C_{i}=2478.5$). Meanwhile, the fixed-effect slope of the trial was significant only for the time taken to draw a side (*Deviation*: $\hat{\beta_{t}^{d}}=-0.001$, SE= 0.004, t=−0.278, p=.800; *Time*: $\hat{\beta_{t}^{t}}=-0.395$, SE= 0.086, t=−4.592, p=0.005). The average relationship as the fixed effect indicated that the triads drew the triangle quickly through trials while maintaining a certain degree of pen deviation.

**Table 2:** Relationship between each task performance and trial considering the variabilities between triads and sides.

Dependent variable	Fixed effect								Random effects			
									Triad		Side	
	Slope				Intercept				Slope’s variance	Intercept’s variance	Slope’s variance	Intercept’s variance
	$\hat{\beta }$	*SE*	*t*-value	*p*-value	$\hat{\beta }$	*SE*	*t*-value	*p*-value				
*Deviation*	−0.001	0.004	−0.278	0.800	0.733	0.201	3.645	0.057	<0.0001	0.015	<0.0001	0.112
*Time*	**−0.395**	**0.086**	**−4.592**	**0.005**	24.042	1.921	12.516	<0.001	0.025	5.300	0.007	7.912

Independent variable is the trial. *Deviation* is the degree of pen deviation on a side (cm) and *Time* is the time taken to draw a side (s). The AIC values are 36.453 and 2464.3 in *Deviation* and *Times’* models.For both performance cases, the full models are selected as well-fitting, where random effects are set in the slope and intercept of a regression (see the Statistical Modeling section).

### Estimation of relationships between improved task performance and role actions

To estimate the relationships between improved task performance through trials and role actions, the index of the time taken to draw a side (s) was regarded as the dependent variable.

**Figure 6:**
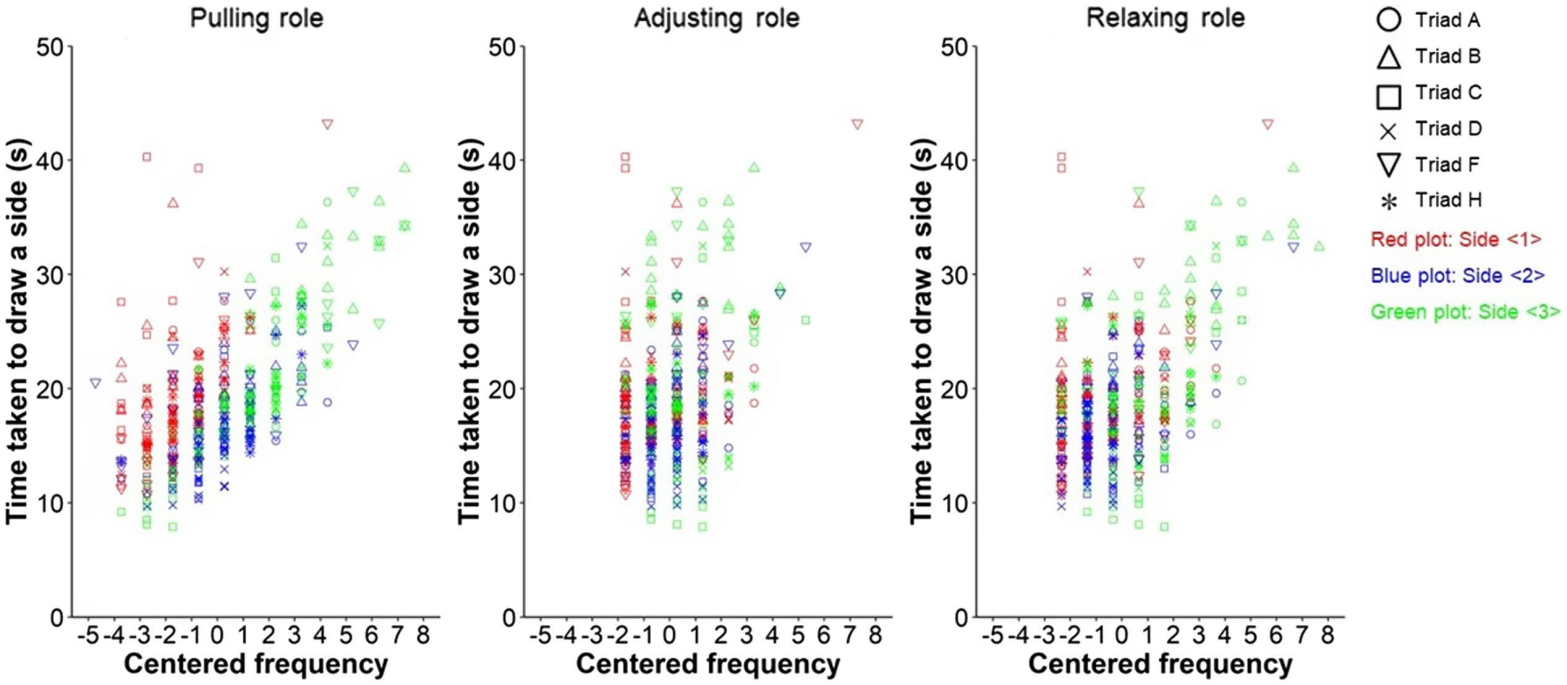
Scatter plots between improved task performance through trials and tension peak frequencies in the pulling, adjusting, and relaxing roles, respectively. The frequencies in the horizontal axes are centered on the averages (see the “Statistical Modeling” section).

Figure 6 shows the scatter plot, in which the vertical and horizontal axes are the time taken to draw a side and the peak frequency in each role. The latter is centered on the average (see the Statistical Modeling section). Table 3 presents the estimated relationship in the well-fitting model (Supplementary material). The full model was selected by comparing the AIC values with the intercept model when the peak frequencies in the three roles were the independent variables $\left(\mathrm{AI}C_{f}=2352.6,\mathrm{AI}C_{i}=2363.5\right)$. In the full model, no variables were excluded based on the VIF values (Full: $VIF_{f}^{p}=$1.258, $VIF_{f}^{a}=$1.686, $VIF_{f}^{r}=$1.428; Intercept: $VIF_{i}^{p}=$ 1.291, $VIF_{i}^{a}=$ 2.068, $VIF_{i}^{r}=$ 2.326). Only the fixed-effect slope of the pulling role was significant (Pull: $\hat{\beta_{f}^{p}}=1.692$, SE= 0.328, t=5.160, p=.022; Adjust: $\hat{\beta_{f}^{a}}=0.441$, SE= 0.450, t=0.979, p=.367; Relax: $\hat{\beta_{f}^{r}}=0.041$, SE= 0.326, t=0.125, p=.912). The average relationship as the fixed effect explained that a lower peak frequency in this role was related to the faster drawing on a side.

**Table 3:** Relationship between improved task performance through trials and tension peak frequencies in the pulling and adjusting, and relaxing roles considering the variabilities between triads and sides.

Independent variables	Fixed effects								Random effects			
									Triad		Side	
	Slope				Intercept				Slope’s variance	Intercept’s variance	Slope’s variance	Intercept’s variance
	$\hat{\beta }$	*SE*	*t*-value	*p*-value	$\hat{\beta }$	*SE*	*t*-value	*p*-value				
*Pulling*	**1.692**	**0.328**	**5.160**	**0.022**					0.050		0.252	
*Adjusting*	0.441	0.450	0.979	0.367	18.728	1.753	10.682	0.004	0.830	2.162	0.084	8.000
*Relaxing*	0.041	0.326	0.125	0.912					0.030		0.232	

Dependent variable is the index of the time taken to draw a side (s). *Pulling*, *Adjusting*, and *Relaxing* are the centered frequencies of tension peaks in the pulling, adjusting, and relaxing roles. The AIC value is 2352.6.The full model is selected as well-fitting, where random effects are set in the slope and intercept of a regression (see the Statistical Modeling section).The bold values in all the tables indicate the crucial points to verify the hypothesis.

**Figure 7:**
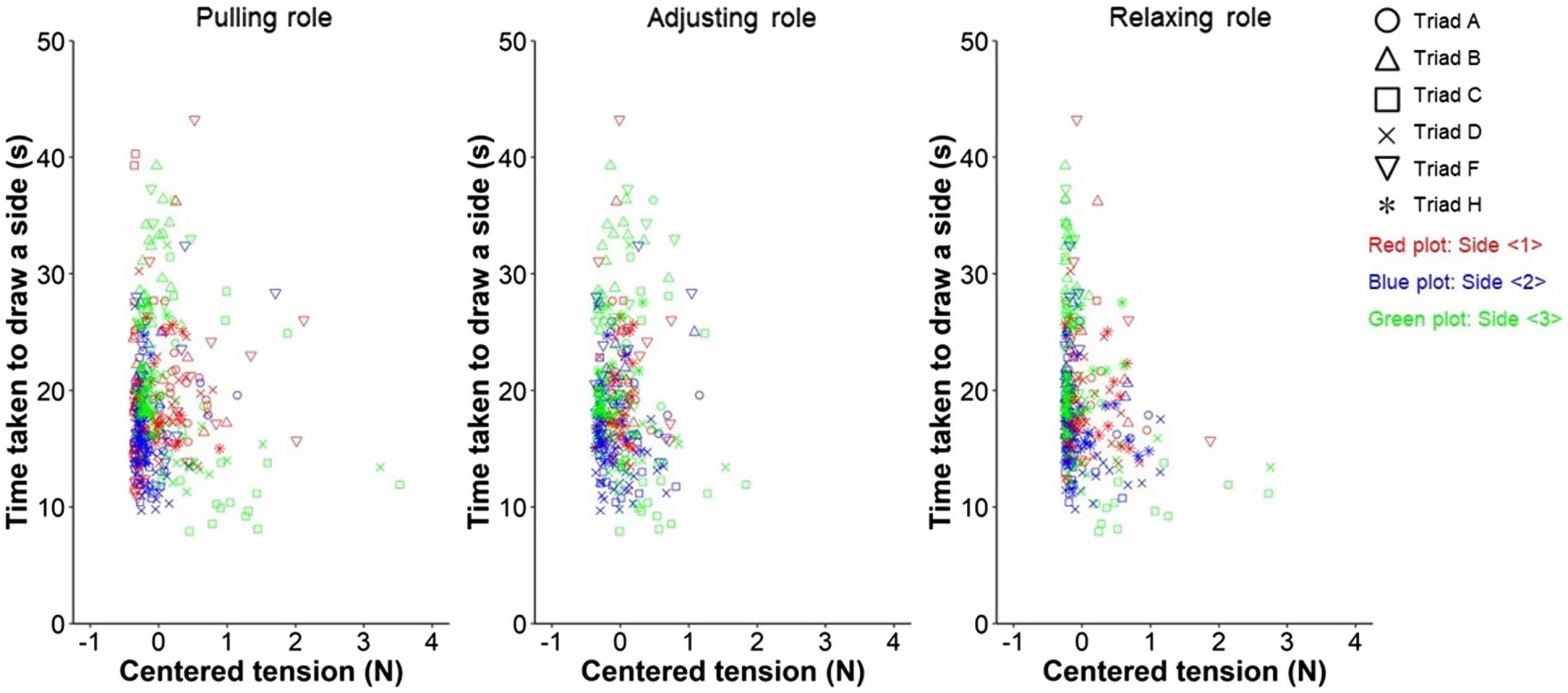
Scatter plots between improved task performance through trials and tension peak values in the pulling, adjusting, and relaxing roles, respectively. The values in the horizontal axes are centered on the averages (see “Statistical Modeling” section).

Meanwhile, Figure 7 shows the scatter plot, in which the vertical and horizontal axes are the time taken to draw a side and the peak value in each role. The latter is centered on the average. Here, the variable of the adjusting role was excluded based on the VIF value ($VIF_{f}^{p}=$ 8.391, $VIF_{f}^{a}=$ 10.446, $VIF_{f}^{r}=$ 2.002), and the full model was corrected (Supplementary code 2). Table 4 represents the estimated relationship in the well-fitting model. The intercept model was selected by comparing the AIC values with the corrected full model $\left(\mathrm{AI}C_{i}=1926.9,\mathrm{AI}C_{cf}=2140.7\right)\text{.}$ In the intercept model, no variables were excluded based on the VIF values (Intercept: $VIF_{i}^{p}=$ 2.413, $VIF_{i}^{a}=$ 2.147, $VIF_{i}^{r}=$1.460; Corrected full: $VIF_{cf}^{p}$= 1.677, $VIF_{cf}^{r}=$1.677). The fixed-effect slopes of the adjusting and relaxing roles were significant (Pull: $\hat{\beta_{v}^{p}}=-1.475$, SE= 0.932, t=−1.583, p=.115; Adjust: $\hat{\beta_{v}^{a}}=2.943\text{,}$ SE= 1.221, t=2.411, p=.016; Relax: $\hat{\beta_{v}^{r}}=-2.832$, SE= 0.793, t=−3.573, p<0.001). The average relationship as the fixed effects explained that a smaller tension peak value in the adjusting role and a larger one of minimum peaks in the relaxing role were related to the faster drawing on a side.

**Table 4:** Relationship between improved task performance through trials and tension peak values in the pulling and adjusting, and relaxing roles considering the variabilities between triads and sides.

Independent variables	Fixed effects								Random effects	
									Triad	Side
	Slope				Intercept					
	$\hat{\beta }$	*SE*	*t*-value	*p*-value	$\hat{\beta }$	*SE*	*t*-value	*p*-value		
*Pulling*	−1.475	0.932	−1.583	0.115						
*Adjusting*	**2.943**	**1.221**	**2.411**	**0.016**	19.804	1.854	10.682	**<**0.001	12.329	3.886
*Relaxing*	**−2.832**	**0.793**	**−3.573**	**<0.001**						

Dependent variable is the index of the time taken to draw a side (s). *Pulling*, *Adjusting*, and *Relaxing* are the centered values of tension peaks in the pulling, adjusting, and relaxing roles. The AIC value is 1926.9.The intercept model is selected as well-fitting, where random effects are set in the intercept of a regression (see the Statistical Modeling section).The bold values in all the tables indicate the crucial points to verify the hypothesis.

Regarding the pulling and relaxing roles, these results suggested that both roles were related to task performance; however, they were predictable considering the task specification. The characteristic of a lower frequency of tension peaks in the pulling role suggested that the participant might smoothly operate the reel and moved the pen. Moreover, the relaxing role was required to support such pen’s movement. Although it is difficult to interpret the feature of a larger value of minimum tension peaks in the relaxing role, something to support the pen’s movement might influence performance. If the pulling and relaxing roles are strongly coupled, multicollinearity may occur; however, it was not confirmed. Meanwhile, notably, it was estimated that the adjusting role, which needed to flexibly change the tension, was related to higher performance of the faster drawing on a side. This result supported our hypothesis that the role to maintain overall balance as a facilitator was crucial for non-verbal coordinated behavior in a triad. Next, we discuss the details of this adjusting-role action.

## Discussion

The results of this study confirmed that the action in the adjusting role was related to the faster drawing on a triangle’s side in a coordinated drawing task. A smaller tension peak value in this role was higher task performance. Here, it should be noted that this operator must generate the tension at least because the pulling and relaxing roles alone result in great pen deviation. According to the task specification and estimated results, the adjusting role might generate the slight tension and intervene in the other roles moderately, such as fine-tuning without disturbing the pen’s movement while avoiding great pen deviation, as shown by dotted magenta and black arrows in Figure 1A. This resilient handling might be crucial for non-verbal coordinated behavior in a triad. The previous sports science study indicated the importance of resilience helping for cooperative and defensive group behavior (Fujii et al., 2016). The function to maintain overall balance as a facilitator would be also required in other non-sports situations, such as learning and debate. Meanwhile, social force, which is the motivation to react to perceived external information including other members, influences coordinated group behavior during a ball possession task in soccer by comparing behavioral experiment and simulation (Yokoyama et al., 2018). Additionally, in a similar task, patients with schizophrenia, who have impairments of visual cognition, impair coordination (Fujii et al., 2020). Furthermore, autistic patients struggle to simulate the positions and situations of other members and adjust one’s own actions (American Psychiatric Association, 1994); it was indicated that higher autistic traits with no cognitive disability lessen flexible actions based on another (Curioni et al., 2017). Based on these findings and our study, fine-tuning actions in coordinated group behavior may be linked to the understanding of other roles. In relation to individual traits mentioned above, Table 1 shows that each participant experienced all the roles in the process of drawing three sides. We established the statistical models including the random effects of both triad and side. The random effect of side would represent the influence of correspondence changes between the participants and roles. The results showed that the time taken to draw a side (s) was, on average, related to the action in the adjusting role, even considering the two random effects. Moreover, we recruited the triads that were as uniform as possible (Participants section). Therefore, in this study, the effects of individual traits, such as perspective-taking ability, and correspondences between the individuals and assigned roles might be small. However, comparing the interaction between triads, among which perspective-taking ability considerably differs, is important for our understanding of complex dynamically planned coordination.

In this study, novice triads significantly improved task performance through trials; they might acquire some skills of balancing overall coordination as the adjusting role, which moderately intervened in other roles and handled based on their actions resiliently. Although there is room for consideration, our findings may correspond to the theories of planned coordination in cognitive science and psychology. Previous studies primarily conducted laboratory experiments, analyzed pair interactions, and confirmed that high performance is achieved through representing other roles, monitoring, and anticipating others’ actions, through sharing and switching roles (e.g., Shirouzu et al., 2002; Sebanz et al., 2003, 2006; Hayashi and Miwa, 2009; Sebanz and Knoblich, 2009). Hence, the novelty and methodological contribution of this study may develop the range of discussion on planned coordination to non-verbal group behavior in a triad. Our discussions of the relationship between improved performance, coordinated group behavior, and role sharing would be meaningful work, as it may elaborate our understanding of social interactions in cognitive science.

However, we could not evaluate group dynamics and roles coupling during the coordinated drawing task directly. It is important to develop ideal indices for discussing complex and dynamically planned coordination itself based on sharing heterogeneous roles as a previous study of biological collective behavior did (Attanasi et al., 2014). This study conducted an observation-based experiment. For instance, another experiment, in which participants draw one side in the condition without intervention by the adjusting role, may be required to investigate the details of the contribution by the adjusting role; we would compare its result with this experiment. However, researchers should remember that the pulling and relaxing roles alone could not draw a side, as shown by the dotted magenta arrow in Figure 1A. Therefore, developing ideal indices for discussing the adjusting and other roles coupling is also necessary using the data in this experiment. Meanwhile, the results in this study alone cannot explain the details of the adjustment process; it is difficult to discuss how the adjusting role changes one’s own actions flexibly using what information of other roles. A multiagent simulation method including machine learning (e.g., Couzin et al., 2002; Fujii et al., 2018) would be effective in solving this problem. For example, the coordinated drawing task is designed by pulling the thread and moving the pen. We can present the adjustment process using force-based models. In the future, we will focus on the adjusting role and introduce the simulation method; this approach will supplement the experimental results of this study. We plan to formulate the three heterogeneous roles using equations of motion and investigate how their parameters affect task performance. Through comparing the results of this study and simulation, further understanding of cognitive processing that underlies fine-tuning actions to maintain overall balance is expected.

## Conclusion

This study used a coordinated drawing task in a triad to analyze non-verbal group behavior based on sharing heterogeneous roles, and statistically estimated the average relationships between task performance and trial, and improved performance and role actions. The results showed that the action in the adjusting role was related to faster drawing on a side. It might moderately intervene in the pulling and relaxing roles and maintain overall balance through fine-tuning without disturbing the pen’s movement while avoiding great pen deviation. This role as a facilitator with resilient handling might be crucial in complex and dynamically planned coordination. In the future, we will focus on the adjusting role and introduce a simulation method to understand such action constructively.

## Data availability statement

The datasets presented in this study can be found in online repositories. The names of the repository/repositories and accession number(s) can be found in the article/Supplementary material.

## Ethics statement

This study was approved by the ethics and safety committee of Kanagawa University, where the experiment was conducted and where the first author was affiliated at that time. The patients/participants provided their written informed consent to participate in this study. Written informed consent was obtained from the individual(s) for the publication of any potentially identifiable images or data included in this article.

## Author contributions

JI conceived the original concept of the investigation of coordinated behavior based on sharing heterogeneous roles in a triad, designed and conducted the experiment, analyzed the data, and wrote the paper. KF provided critical comments during research processes. All authors contributed to the article and approved the submitted version.

## Funding

This study was supported by JSPS KAKENHI Grant numbers 19 K24369, 21 K18033, and 18 K18116.

## Conflict of interest

The authors declare that the research was conducted in the absence of any commercial or financial relationships that could be construed as a potential conflict of interest.

## Publisher’s note

All claims expressed in this article are solely those of the authors and do not necessarily represent those of their affiliated organizations, or those of the publisher, the editors and the reviewers. Any product that may be evaluated in this article, or claim that may be made by its manufacturer, is not guaranteed or endorsed by the publisher.

## Footnotes

^1^The origin source of some explanations in this section refers to our proceedings paper (Ichikawa and Fujii, 2021).

## References

[ref1] American Psychiatric Association (1994). Diagnostic and Statistical Manual of Mental Disorders (DSM-IV). 4th Edn. eds. Ross R. Davis W. (Washington, DC: American Psychiatric Association).

[ref2] Amici F. Widdig A. (2019). An evolutionary perspective on the development of primate sociality. Behav. Ecol. Sociobiol. 73:116. doi: 10.1007/s00265-019-2722-8

[ref3] Araújo D. Davids K. (2016). Team synergies in sport: theory and measures. Front. Psychol. 7:1449. doi: 10.3389/fpsyg.2016.01449, PMID: 27708609 PMC5030782

[ref4] Attanasi A. Cavagna A. Del Castello L. Giardina I. Melillo S. Parisi L.. (2014). Collective behaviour without collective order in wild swarms of midges. PLoS Comput. Biol. 10:e1003697. doi: 10.1371/journal.pcbi.1003697, PMID: 25057853 PMC4109845

[ref5] Battocchi A. Gal E. Sasson A. B. Pianesi F. Venuti P. Zancanaro M.. (2008). “Collaborative puzzle game-an interface for studying collaboration and social interaction for children who are typically developed or who have autistic Spectrum disorder,” in ICDVRAT 2008: Proceedings of the 7th International Conference on Disability, Virtual Reality and Associated Technologies, With Artabilitation 2008 Sep 8-11; Maia, Portugal. eds. Sharkey P. Lopes-dos-Santos P. Weiss P. L. Brooks T. (Reading, UK: The University of Reading), 127–134.

[ref6] Berta W. Cranley L. Dearing J. W. Dogherty E. J. Squires J. E. Estabrooks C. A. (2015). Why (we think) facilitation works: insights from organizational learning theory. Implement. Sci. 10:141. doi: 10.1186/s13012-015-0323-0, PMID: 26443999 PMC4596304

[ref7] Böckler A. Knoblich G. Sebanz N. (2012). Effects of a coactor's focus of attention on task performance. J. Exp. Psychol. Hum. Percept. Perform. 38, 1404–1415. doi: 10.1037/a0027523, PMID: 22409143

[ref8] Boonman A. Fenton B. Yovel Y. (2019). The benefits of insect-swarm hunting to echolocating bats, and its influence on the evolution of bat echolocation signals. PLoS Comput. Biol. 15:e1006873. doi: 10.1371/journal.pcbi.1006873, PMID: 31830029 PMC6907744

[ref9] Braun D. A. Ortega P. A. Wolpert D. M. (2009). Nash equilibria in multi-agent motor interactions. PLoS Comput. Biol. 5:e1000468. doi: 10.1371/journal.pcbi.1000468, PMID: 19680426 PMC2714462

[ref10] Brown V. A. (2021). An introduction to linear mixed-effects modeling in R. Adv. Meth. Pract. Psychol. Sci. 4. doi: 10.1177/2515245920960351

[ref11] Chackochan V. T. Sanguineti V. (2019). Incomplete information about the partner affects the development of collaborative strategies in joint action. PLoS Comput. Biol. 15:e1006385. doi: 10.1371/journal.pcbi.1006385, PMID: 31830100 PMC6907753

[ref12] Couzin I. D. Krause J. James R. Ruxton G. D. Franks N. R. (2002). Collective memory and spatial sorting in animal groups. J. Theor. Biol. 218, 1–11. doi: 10.1006/jtbi.2002.3065, PMID: 12297066

[ref13] Curioni A. Minio-Paluello I. Sacheli L. M. Candidi M. Aglioti S. M. (2017). Autistic traits affect interpersonal motor coordination by modulating strategic use of role-based behavior. Mol. Autism. 8:23. doi: 10.1186/s13229-017-0141-0, PMID: 28616126 PMC5466762

[ref14] De Jaegher H. Di Paolo E. Gallagher S. (2010). Can social interaction constitute social cognition? Trends Cogn. Sci. 14, 441–447. doi: 10.1016/j.tics.2010.06.009, PMID: 20674467

[ref15] Evans M. A. Feenstra E. Ryon E. McNeill D. (2011). A multimodal approach to coding discourse: collaboration, distributed cognition, and geometric reasoning. Int. J. Comput. Support Collab. Learn. 6, 253–278. doi: 10.1007/s11412-011-9113-0

[ref16] Fujii K. Kawasaki T. Inaba Y. Kawahara Y. (2018). Prediction and classification in equation-free collective motion dynamics. PLoS Comput. Biol. 14:e1006545. doi: 10.1371/journal.pcbi.1006545, PMID: 30395600 PMC6237418

[ref17] Fujii K. Yokoyama K. Koyama T. Rikukawa A. Yamada H. Yamamoto Y. (2016). Resilient help to switch and overlap hierarchical subsystems in a small human group. Sci. Rep. 6:23911. doi: 10.1038/srep23911, PMID: 27045443 PMC4820690

[ref18] Fujii K. Yoshihara Y. Matsumoto Y. Tose K. Takeuchi H. Isobe M.. (2020). Cognition and interpersonal coordination of patients with schizophrenia who have sports habits. PLoS One 15:e0241863. doi: 10.1371/journal.pone.0241863, PMID: 33166326 PMC7652240

[ref21] Hayashi Y. (2018). The power of a “maverick” in collaborative problem solving: an experimental investigation of individual perspective-taking within a group. Cogn. Sci. 42, 69–104. doi: 10.1111/cogs.12587, PMID: 29388252

[ref22] Hayashi Y. Miwa K. (2009). “Prior experience and communication media in establishing common ground during collaboration.” in Proceedings of the 31st Annual Meeting of the Cognitive Science Society; 2009 July 29-Aug 1; Amsterdam, Netherlands. eds. Taatgen N. A. H. van Rijn (Austin, TX: Cognitive Science Society), 526–531.

[ref23] Himichi T. Osanai H. Goto T. Fujita H. Kawamura Y. Davis M. H.. (2017). Development of a Japanese version of the interpersonal reactivity index. Jpn. J. Psychol. 88, 61–71. doi: 10.4992/jjpsy.88.15218, PMID: 29630312

[ref24] Hutchins E. (1995). Cognition in the Wild. ed. Hutchins E. (Cambridge: MIT Press).

[ref25] Ichikawa J. Fujii K. (2020). Proposal of a research approach for discussion of a dynamic coordination mechanism: investigation of anticipating others’ behaviors and adaptation through quantitative analysis of group behavior. Cogn. Stud. Bull. Jpn. Cogn. Sci. Soc. 27, 377–385. doi: 10.11225/cs.2020.026

[ref26] Ichikawa J. Fujii K. (2021). “Understanding others’ roles based on perspective taking in coordinated group behavior,” in CogSci 2021: Proceedings of the 43rd Annual Meeting of the Cognitive Science Society; 2021 July 27-30; Online. eds. Fitch T. Lamm C. Leder H. Raible K. T. (Austin, TX: Cognitive Science Society), 1285–1291.

[ref27] Ichikawa J. Fujii K. Nagai T. Omori T. Oka N. (2021). Quantitative analysis of spontaneous sociality in children’s group behavior during nursery activity. PLoS One 16:e0246041. doi: 10.1371/journal.pone.0246041, PMID: 33529267 PMC7853442

[ref19] Kelso J. A. (2021). Unifying large-and small-scale theories of coordination. Entropy 23:537. doi: 10.3390/e23050537, PMID: 33925736 PMC8146522

[ref20] Knoblich G. Butterfill S. Sebanz N. (2011). Psychological research on joint action: theory and data. Psychol. Learn. Motiv. 54, 59–101. doi: 10.1016/B978-0-12-385527-5.00003-6

[ref28] Leppink J. (2018). Analysis of covariance (ANCOVA) vs. moderated regression (MODREG): why the interaction matters. Health Prof. Educ. 4, 225–232. doi: 10.1016/j.hpe.2018.04.001

[ref29] Lessard S. Bareil C. Lalonde L. Duhamel F. Hudon E. Goudreau J.. (2015). External facilitators and interprofessional facilitation teams: a qualitative study of their roles in supporting practice change. Implement. Sci. 11:97. doi: 10.1186/s13012-016-0458-7, PMID: 27424171 PMC4947272

[ref30] Maruno S. (1991). Effects of social interaction on preschool children’s acquisition of procedural knowledge and “self-other perspectives coordination”. Jpn. J. Devep. Dev. Psychol. 1, 116–127. doi: 10.11201/jjdp.1.116

[ref31] Michael J. McEllin L. Felber A. (2020). Prosocial effects of coordination: what, how and why? Acta Psychol. 207:03083. doi: 10.1016/j.actpsy.2020.103083, PMID: 32422420

[ref32] Nalepka P. Silva P. L. Kallen R. W. Shockley K. Chemero A. Saltzman E.. (2021). Task dynamics define the contextual emergence of human corralling behaviors. PLoS One 16:e0260046. doi: 10.1371/journal.pone.0260046, PMID: 34780559 PMC8592491

[ref33] Richardson M. J. Marsh K. L. Isenhower R. W. Goodman J. R. Schmidt R. C. (2007). Rocking together: dynamics of intentional and unintentional interpersonal coordination. Hum. Mov. Sci. 26, 867–891. doi: 10.1016/j.humov.2007.07.002, PMID: 17765345

[ref34] Riley M. A. Richardson M. Shockley K. Ramenzoni V. C. (2011). Interpersonal synergies. Front. Psychol. 2:38. doi: 10.3389/fpsyg.2011.00038, PMID: 21716606 PMC3110940

[ref35] Schmidt R. C. Carello C. Turvey M. T. (1990). Phase transitions and critical fluctuations in the visual coordination of rhythmic movements between people. J. Exp. Psychol. Hum. Percept. Perform. 16, 227–247. doi: 10.1037/0096-1523.16.2.227, PMID: 2142196

[ref36] Sebanz N. Bekkering H. Knoblich G. (2006). Joint action: bodies and minds moving together. Trends Cogn. Sci. 10, 70–76. doi: 10.1016/j.tics.2005.12.009, PMID: 16406326

[ref37] Sebanz N. Knoblich G. Prinz W. (2003). Representing others’ actions: just like one’s own? Cognition 88, B11–B21. doi: 10.1016/S0010-0277(03)00043-X, PMID: 12804818

[ref38] Sebanz N. Knoblich G. (2009). Prediction in joint action: what, when, and where. Top. Cogn. Sci. 1, 353–367. doi: 10.1111/j.1756-8765.2009.01024.x 25164938

[ref39] Shirouzu H. Miyake N. Masukawa H. (2002). Cognitively active externalization for situated reflection. Cog. Sci. 26, 469–501. doi: 10.1207/s15516709cog2604_3

[ref40] Steiner S. Macquet A. C. Seiler R. (2017). An integrative perspective on interpersonal coordination in interactive team sports. Front. Psychol. 8:1440. doi: 10.3389/fpsyg.2017.01440, PMID: 28894428 PMC5581343

[ref41] Takagi A. Ganesh G. Yoshioka T. Kawato M. Burdet E. (2017). Physically interacting individuals estimate the partner’s goal to enhance their movements. Nat. Hum. Behav. 1:0054. doi: 10.1038/s41562-017-0054

[ref42] Takagi A. Hirashima M. Nozaki D. Burdet E. (2019). Individuals physically interacting in a group rapidly coordinate their movement by estimating the collective goal. eLife 8:e41328. doi: 10.7554/eLife.41328.001, PMID: 30744805 PMC6372281

[ref43] Tunstrøm K. Katz Y. Ioannou C. C. Huepe C. Lutz M. J. Couzin I. D. (2013). Collective states, multistability and transitional behavior in schooling fish. PLoS Comput. Biol. 9:e1002915. doi: 10.1371/journal.pcbi.1002915, PMID: 23468605 PMC3585391

[ref44] van der Wel R. P. Becchio C. Curioni A. Wolf T. (2021). Understanding joint action: current theoretical and empirical approaches. Acta Psychol. 215:103285. doi: 10.1016/j.actpsy.2021.103285, PMID: 33676068

[ref45] Yokoyama K. Yamamoto Y. (2011). Three people can synchronize as coupled oscillators during sports activities. PLoS Comput. Biol. 7:e1002181. doi: 10.1371/journal.pcbi.1002181, PMID: 21998570 PMC3188505

[ref46] Yokoyama K. Shima H. Fujii K. Tabuchi N. Yamamoto Y. (2018). Social forces for team coordination in ball possession game. Phys. Rev. E 97:022410. doi: 10.1103/PhysRevE.97.022410 29548247

[ref47] Zhu H. (2021). Role-Based Collaboration: Modelling and Solving Problems in the Complex World. ed. Zhu H. (New Jersey: Wiley-IEEE Press).

[ref48] Zhu H. Zhou M. C. (2006). Role-based collaboration and its kernel mechanisms. IEEE Trans. Syst. Man Cybern. Syst. 36, 578–589. doi: 10.1109/TSMCC.2006.875726

[ref49] Zuur A. F. Ieno E. N. Elphick C. S. (2010). A protocol for data exploration to avoid common statistical problems. Methods Ecol. Evol. 1, 3–14. doi: 10.1111/j.2041-210X.2009.00001.x

