# Supplementary material for: Analysis of group behavior based on sharing heterogeneous roles in a triad using a coordinated drawing task
Source: Front Psychol. 2022 Nov 15;13:890205. doi: 10.3389/fpsyg.2022.890205 (PMC9707694; doi:10.3389/fpsyg.2022.890205)
Supplement: Supplementary file 1 [file Data_Sheet_1.zip › Supplementary files/Supplementary Material.pdf]

*Supplementary Material*

# Analysis of group behavior based on sharing heterogeneous roles in a triad using a coordinated drawing task

**Jun Ichikawa*, Keisuke Fujii**

*** Correspondence:** Jun Ichikawa: j-ichikawa@inf.shizuoka.ac.jp

## 1 Measurement Errors of Pen Positions and Tensions

In the experiment, we measured the pen positions in two dimensions and three thread tensions in one dimension during a coordinated drawing task (see Figure 1 in the manuscript). For reference, we used motion analysis software (DITECT Co., Ltd., DIPP-Motion V/2D ver. 1.2.5) and calculated the measurement errors of the pen positions. The average of the absolute errors through the participant triads was 0.000 (cm) × 0.000 (cm). It was very small, at the level of a fourth decimal place or less. We also confirmed the nonlinearity and hysteresis rates of the tension sensors (Tokushukeisoku Co., Ltd., TK-440-01 in the TK-A-30N type). The averages were 0.087% (*SD* = 0.074) and 0.080% (*SD* = 0.000), respectively, which were below 0.1%; there would be no major problems in the measurement.

## 2 Investigation of Pseudo Correlations in Time-series Data of Task Performance through Trials[1]

Before the regression analysis, we investigated pseudo correlations using the time-series data of task performance: (1) the degree of pen deviation on a side (cm) and (2) the time taken to draw a side (s) through trials (see the Task Performance section in the manuscript). Augmented Dickey-Fuller (ADF), Point Optimal (PO), and Durbin-Watson tests were conducted on the data at the 5% level with R-3.6.1, using the tseries 0.10-48, urca 1.3-0, and lmtest 0.9-38 packages.

There were 18 time-series data of each performance index based on a combination of six triads and three sides. The results showed that all the data for the degree of pen deviation on a side and 15 data for the time taken to draw a side were significantly unit root and cointegration, or neither unit root nor autocorrelation of the residuals (see the Supplementary Statistical Results). It was suggested that the pseudo correlations between performance and trial were not present in most of the limited experimental data. Hence, we conducted a regression analysis to estimate the relationship between each performance and trial using all the time-series data (see the Estimation of Relationships between Task Performance and Trial section in the manuscript).

## 3 Non-selected Models as Well-fitting

This study analyzed group behavior during the coordinated drawing task. We referred to a tutorial paper (Brown, 2021) and estimated the relationships between: (1) task performance and trial, and (2)

[1] The origin source of the explanation way in this section refers to our proceedings paper (Ichikawa & Fujii, 2021).

improved performance and three-role actions, using a linear mixed model (see the Statistical Modeling section in the manuscript). In these analysis procedures, non-selected models were statistically less fit than selected ones, and these AIC values were relatively small. Meanwhile, there was a corrected model whose variable was excluded before estimation because the VIF value was greater than the criterion 10 (Zuur et al., 2010).

The AIC and VIF values of the non-selected models as well-fitting are summarized in the Supplementary Statistical Results.

## 4 Disclosure of Prepared Data

The dataset used, R analysis codes, and a sample movie of the experiment are provided in the supplementary files.

The dataset has a structure of four variables, which were recorded in each trial, across the six triads and three sides. The variables are the degree of pen deviation on a side (cm), the time taken to draw a side (s), the number of counts (defined as peak frequency) recorded tension peaks, and the peak value (N) (see the Role Actions section in the manuscript). In the dataset, variables labeled “Deviation” and “Time” show task performance mentioned above. Variables labeled “FreqPull,” “FreqAdjust,” and “FreqRelax” indicate the peak frequencies in the pulling, adjusting, and relaxing roles. “TensionPull” and “TensionAdjust” are the average tension peaks (N) defined as peak values; meanwhile, “TensionRelax” is the minimum tension peak (N). A null value represents missing data due to measurement problems, such as thread breaking, or indicates that the tension peaks were not extracted on a side in each role. The six triads engaged in the experimental task repeatedly for about 25 trials in which three sides were drawn. There are approximately 450 samples in each variable based on a combination of six triads, 25 trials, and three side. Additionally, we prepared three R codes to investigate the relationships between: (1) task performance and trial, and (2) improved performance and three-role actions, and (3) the effects of the triad and side factors on the tension peak values in each role. In these codes, the demo scripts and licenses are mentioned. We also provided the sample movie of the coordinated drawing task to help understanding the task difficulty and group dynamics. It shows three representative trials at the beginning, middle, and end for triad C.

## Reference

Brown, V. A. (2021). An introduction to linear mixed-effects modeling in R. *Adv. Meth. Pract. Psychol. Sci.* 4. doi:10.1177/2515245920960351

Ichikawa, J., and Fujii, K. (2021). ”Understanding others’ roles based on perspective taking in coordinated group behavior,” in *Proceedings of the 43rd Annual Meeting of the Cognitive Science Society*, eds. T. Fitch, C. Lamm, H. Leder, and K. T. Raible (Austin, TX: Cognitive Science Society), 1285-1291.

Zuur, A. F., Ieno, E. N., and Elphick, C. S. (2010). A protocol for data exploration to avoid common statistical problems. *Methods in Ecology and Evolution*, Vol. 1, No. 1, 3-14. doi:10.1111/j.2041-210X.2009.00001.x
